# Direct regulation of fibroblast growth factor 23 by energy intake through mTOR

**DOI:** 10.1038/s41598-020-58663-7

**Published:** 2020-02-04

**Authors:** Angela Vidal, Rafael Rios, Carmen Pineda, Ignacio Lopez, Juan R. Muñoz-Castañeda, Mariano Rodriguez, Escolastico Aguilera-Tejero, Ana I. Raya

**Affiliations:** 1 Department of Animal Medicine and Surgery, University of Cordoba, Campus Universitario Rabanales, Cordoba, Spain; 2 Maimonides Biomedical Research Institute of Cordoba (IMIBIC), Reina Sofia University Hospital, University of Cordoba, Cordoba, Spain

**Keywords:** Endocrinology, Endocrine system and metabolic diseases

## Abstract

To test the hypothesis that fibroblast growth factor 23 (FGF23) is directly regulated by energy intake, *in vivo* and *in vitro* experiments were conducted. Three groups of rats were fed diets with high (HC), normal (NC) and low (LC) caloric content that resulted in different energy intake. *In vitro*, UMR106 cells were incubated in high (HG, 4.5 g/l) or low glucose (LG, 1 g/l) medium. Additional treatments included phosphorus (P), mannitol, rapamycin and everolimus. Intestinal absorption of P and plasma P concentrations were similar in the three groups of rats. As compared with NC, plasma FGF23 concentrations were increased in HC and decreased in the LC group. A significant correlation between energy intake and plasma FGF23 concentrations was observed. *In vitro*, mRNA FGF23 was significantly higher in UMR106 cells cultured in HG than in LG. When exposed to high P, mRNA FGF23 increased but only when cells were cultured in HG. Cells incubated with HG and mechanistic target of rapamycin (mTOR) inhibitors expressed low mRNA FGF23, similar to the values obtained in LG. In conclusion, this study shows a direct regulation of FGF23 production by energy availability and demonstrates that the mTOR signaling pathway plays a central role in this regulatory system.

## Introduction

Fibroblast growth factor 23 (FGF23) is a phosphaturic hormone secreted by osteocytes/osteoblasts that plays a major role in the regulation of mineral metabolism^1^. The interaction of FGF23 with its receptor, FGFR1c, and its co-receptor, klotho, produces two distinct effects in the kidneys: (a) FGF23 increases phosphaturia by inducing internalization of the sodium-dependent phosphate (P) transporters (NaPi2a and NaPi2c)^2^; and, (b) FGF23 modulates vitamin D metabolism through downregulation of 1-α-hydroxylase (CYP27B1) and upregulation of 24-hydroxylase (CYP24A1), thus leading to a decrease in circulating calcitriol concentrations^2^.

Increased levels of FGF23, usually found in patients with renal failure, have been reported as a risk factor of cardiovascular mortality^3^. The relationship between high FGF23 and mortality is not restricted to patients with kidney disease but has also been identified in the general population^4,5^.

Synthesis and secretion of FGF23 is regulated mainly by dietary P and increased P balance is thought to be the main stimulus for FGF23 secretion^6^. In addition, FGF23 production is under hormonal control by calcitriol, that decreases FGF23 synthesis^7^. Moreover, parathyroid hormone^8^, inflammation^9^ and iron deficiency^10^ have been reported to influence FGF23 production.

Epidemiological studies have suggested that FGF23 might be regulated by energy intake. FGF23 has been shown to be elevated in obese people^11^ and increased energy intake has been identified as a potential predictor of plasma FGF23 concentrations^12^. In addition, metabolites involved in the regulation of energy metabolism have been recently reported to regulate FGF23 production, although the results are somewhat contradictory since increases in both insulin, that is typically associated to energy repletion, and AMP-activated kinase (AMPK), which is activated by energy depletion, have been reported to decrease FGF23 synthesis^13,14^.

Experimental work has shown that feeding hypercaloric diets to rodents results in a consistent increase in plasma and bone FGF23^15,16^. In these studies, energy density of food was increased by elevating the fat content of the diet. In addition to the effect of increased caloric intake, high fat feeding may elicit systemic inflammation^17^ and renal injury^18^ which could also influence FGF23^19^, thus a direct relationship between energy intake and FGF23 has not been clearly established.

The mechanistic (previously mammalian) target of rapamycin (mTOR) pathway controls cellular signaling in response to nutrients and mitogens, and inhibition of mTOR has been shown to mimic caloric restriction^20^. mTOR is a protein kinase that belongs to the phosphoinositide 3-kinase (PI3K)-related kinase family and interacts with several proteins to form two distinct multiprotein complexes: mTORC1 and mTORC2^21^. Only mTORC1, which is a key sensor of energy availability and nutritional status, is inhibited by rapamycin^22^.

The aim of this work was to test the hypothesis that FGF23 is directly regulated by energy intake. To this purpose we investigated, *in vivo*, the effect of increased and reduced energy intake on FGF23. In addition, the effect of energy availability on FGF23 production by bone cells was studied *in vitro* and the involvement of the mTOR signaling pathway in the regulation of FGF23 production was evaluated.

## Results

### *In vivo* studies

**Figure 1:**
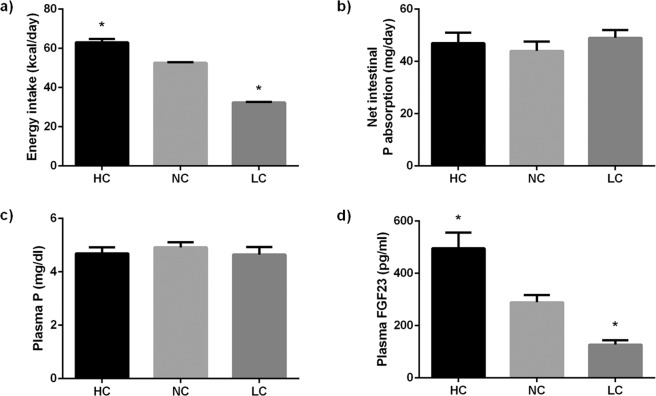
Energy intake (**a**), net intestinal absorption of P (**b**), plasma P concentrations (**c**) and plasma FGF23 concentrations (**d**) in rats (n = 9 per group) fed diets with a high (HC), normal (NC) or low (LC) caloric content. *p < 0.05 vs NC; one-way ANOVA with Fisher LSD post-hoc test.

As expected, energy intake was significantly (p < 0.001) higher in the HC group, 63.0 ± 1.7 kcal/day, than in the NC group, 52.6 ± 0.3 kcal/day. Differences in energy intake in the HC group were not as high as would be predicted from the energy content of the diets because these rats, that were fed ad libitum, voluntarily reduced their food consumption to 12.0 ± 0.3 g/day, when compared with the NC group, 14.9 ± 0.1 g/day. Rats fed LC had their food supply adjusted to 24.7 ± 0.3 g/day and energy intake was much lower (p < 0.0001) in LC rats, 32.4 ± 0.3 kcal/day, than in NC rats (Fig. 1a). Plasma glucose concentrations were not influenced by caloric intake and similar values were obtained in rats fed NC, 124.6 ± 5.7 mg/dl, and LC, 126.9 ± 4.7 mg/dl; rats fed HC had slightly lower glucose concentrations, 113.0 ± 8.3 mg/dl, but the differences with NC and LC were not significant.

Even though all diets had the same P concentration (0.6%), P intake was modulated by food intake. Thus, the HC group ingested slightly less P, 72.2 ± 2.0 mg/day, than the NC group, 89.7 ± 0.6 mg/day, while rats fed LC ingested more P (p < 0.0001) than the other groups, 148.3 ± 1.5 mg/day. To assess whether P absorption was different between groups, fecal P was measured and net intestinal absorption of P was calculated. As shown in Fig. 1b, there were no major differences in the amount of P absorbed between the study groups: NC, 44.0 ± 3.6 mg/day, HC, 47.0 ± 4.0 mg/day, and LC, 49.0 ± 3.0 mg/day.

Plasma P concentration was not influenced by calorie intake and values were slightly lower in the HC group, 4.7 ± 0.2 mg/dl, and in the LC group, 4.7 ± 0.3 mg/dl, than the NC group, 5.0 ± 0.2 mg/dl (Fig. 1c).

**Figure 2:**
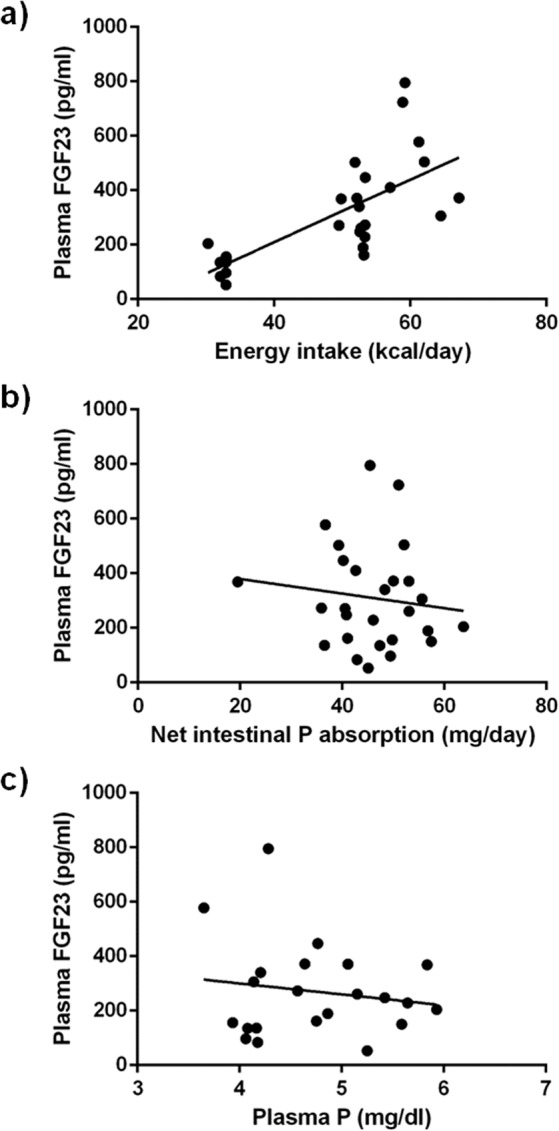
Correlation between: (**a**) plasma FGF23 concentrations and energy intake (r = 0.705, p < 0.0001), (**b**) plasma FGF23 concentrations and net intestinal absorption of P (r = −0.124, p = 0.535), and (**c**) plasma FGF23 and plasma P concentrations (r = −0.154, p = 0.493) in rats fed diets with a high (HC), normal (NC) or low (LC) caloric content. Pearson correlation test.

Plasma FGF23 concentrations in NC rats were 289 ± 28 pg/ml. A significant (p = 0.001) increase in plasma FGF23 was observed in the HC group, 496 ± 60 pg/ml. In contrast, rats in the LC group had lower (p = 0.009) FGF23 concentrations, 127 ± 17 pg/ml, than rats in the NC group (Fig. 1d). A strong correlation between energy intake and plasma FGF23 was observed (r = 0.705, p < 0.0001) (Fig. 2a); however, FGF23 did not correlate with the intestinal absorption of P (r = −0.125, p = 0.535) (Fig. 2b) or with plasma P concentration (r = −0.154, p = 0.493) (Fig. 2c).

**Figure 3:**
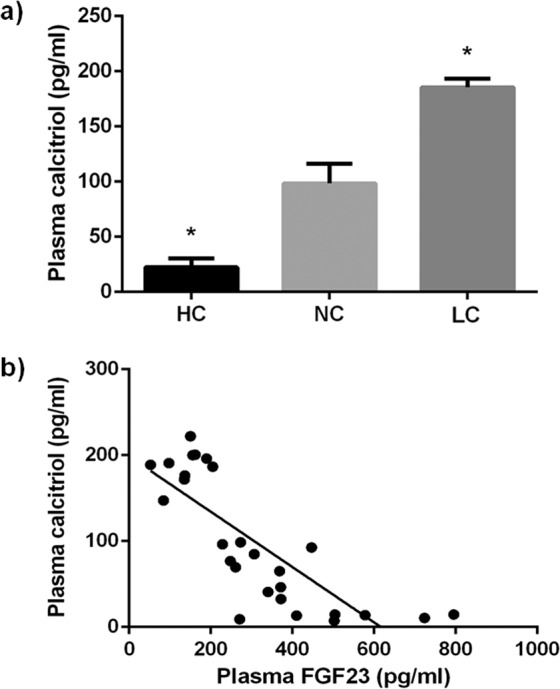
(**a**) Plasma calcitriol concentrations in rats (n = 9 per group) fed diets with a high (HC), normal (NC) or low (LC) caloric content. *p < 0.05 vs NC; one-way ANOVA with Fisher LSD post-hoc test. (**b**) Correlation between plasma FGF23 and plasma calcitriol concentrations (r = −0.803, p < 0.0001) in rats fed diets with a high (HC), normal (NC) or low (LC) caloric content. Pearson correlation test.

As compared with the NC group, 98.4 ± 17.8 pg/ml, plasma calcitriol levels were reduced (p = 0.0003) in the rats fed HC, 22.4 ± 8.2 pg/ml, whereas they were increased in the LC group, 185.6 ± 7.7 pg/ml, (p < 0.0001 vs NC) (Fig. 3a). Plasma calcitriol and FGF23 concentrations were inversely correlated (r = −0.803, p < 0.0001) (Fig. 3b).

### *In vitro* studies

**Figure 4:**
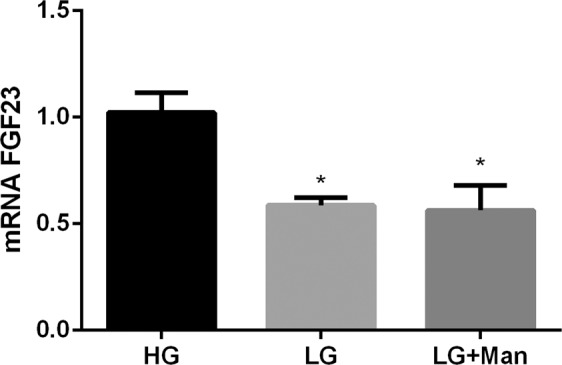
mRNA FGF23 vs Tbp expression (arbitrary units) by UMR 106 cells cultured in medium with high glucose (HG), low glucose (LG) and low glucose + mannitol (LG + Man). *p < 0.05 vs HG; one-way ANOVA with Fisher LSD post-hoc test.

FGF23 mRNA expression was significantly higher (p < 0.0001) in UMR 106 cells cultured for 6 days in HG, 1.02 ± 0.09, than in UMR 106 cells cultured in LG, 0.59 ± 0.03. However, a shorter exposure to LG or a moderate decrease in glucose concentration did not reduce mRNA FGF23 (Suppl. Fig. 1). When osmolality of the LG medium was increased to the same level than the HG medium by adding mannitol, mRNA FGF23 was not affected and remained lower (p = 0.002) in cells incubated in LG + Man, 0.56 ± 0.11, than in cells incubated in HG, 1.02 ± 0.09 (Fig. 4).

**Figure 5:**
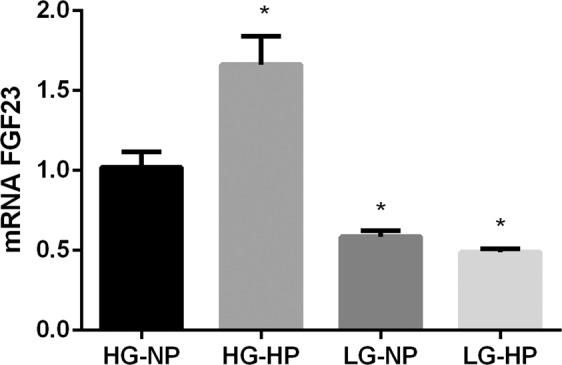
mRNA FGF23 vs Tbp expression (arbitrary units) by UMR 106 cells cultured in medium with high glucose and normal P (HG-NP), high glucose and high P (HG-HP), low glucose and normal P (LG-NP) and low glucose and high P (LG-HP). *p < 0.05 vs HG-NP; one-way ANOVA with Fisher LSD post-hoc test.

UMR 106 cells increased FGF23 mRNA expression when exposed to HP but only if they were cultured in HG, 1.66 ± 0.18 vs 1.02 ± 0.09 (p < 0.0001). However, cells cultured in LG exposed to HP or NP had comparable FGF23 mRNA expression, 0.50 ± 0.02 and 0.59 ± 0.03, respectively (Fig. 5).

**Figure 6:**
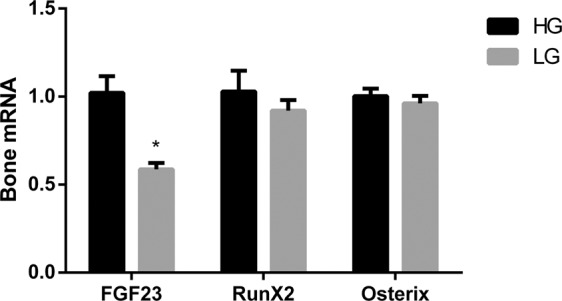
mRNA FGF23 vs Tbp, RunX2 vs Tbp and Osterix vs Tbp expression (arbitrary units) by UMR 106 cells cultured in medium with high glucose (HG, black bars) and low glucose (LG, grey bars). *p < 0.05 vs HG; Student’s t-test.

Expression of osteogenic genes in UMR 106 cells was not modulated by changes in glucose concentration in the culture medium. Thus, neither mRNA RunX2, 1.03 ± 0.12 vs 0.92 ± 0.06, nor mRNA Osterix, 1.00 ± 0.04 vs 0.96 ± 0.04, changed their expression when cultured in either HG or LG (Fig. 6).

**Figure 7:**
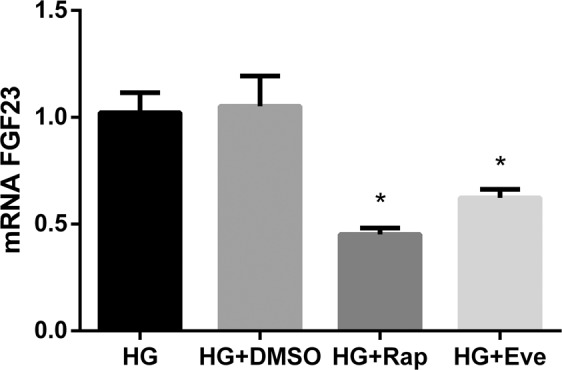
mRNA FGF23 vs Tbp expression (arbitrary units) by UMR 106 cells cultured in medium with high glucose (HG), high glucose + DMSO (HG + DMSO), HG + Rapamycin 10 nM (HG + Rap) and HG + Everolimus 10 nM (HG + Eve). *p < 0.05 vs HG; one-way ANOVA with Fisher LSD post-hoc test.

The addition of rapamycin to UMR 106 cells cultured in HG caused a marked reduction of FGF23 mRNA, from 1.02 ± 0.09 to 0.45 ± 0.03 (p = 0.001), reaching values similar to those observed in cells incubated in LG, 0.59 ± 0.03. Addition of DMSO (rapamycin solvent) to the HG medium did not affect FGF23 mRNA, 1.05 ± 0.14 vs 1.02 ± 0.09 (Fig. 7). Dose response and time response to incubation with rapamycin are shown in Suppl. Fig. 1.

When UMR 106 cells were incubated with another mTOR inhibitor, everolimus, FGF23 mRNA was also reduced, from 1.02 ± 0.09 to 0.62 ± 0.04 (p = 0.005) (Fig. 7).

**Figure 8:**
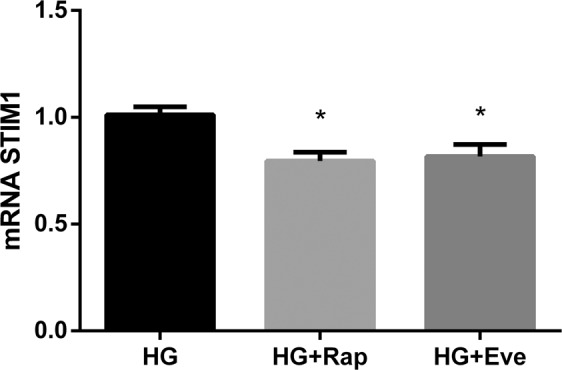
mRNA STIM1 vs Tbp expression (arbitrary units) by UMR 106 cells cultured in medium with high glucose (HG), HG + Rapamycin 10 nM (HG + Rap) and HG + Everolimus 10 nM (HG + Eve). *p < 0.05 vs HG; one-way ANOVA with Fisher LSD post-hoc test.

UMR 106 cells incubated with rapamycin decreased the expression of stromal interaction molecule 1 (STIM1); thus, after treatment with rapamycin, STIM1 mRNA decreased from 1.01 ± 0.05 to 0.79 ± 0.05 (p = 0.004). Moreover, incubation with everolimus also decreased STIM1 expression from 1.01 ± 0.05 to 0.82 ± 0.06 (p = 0.02) (Fig. 8).

## Discussion

This study was designed to investigate the influence of energy intake on FGF23. Our results demonstrate a direct relationship between these two parameters. Thus, *in vivo*, an increase in energy intake was associated to higher FGF23 concentrations and a reduction in energy intake resulted in lower FGF23 levels. In addition, *in vitro*, energy availability also modulated FGF23 expression by bone cells, which decreased when the glucose concentration in the culture medium was reduced. Moreover, in *vitro* studies indicate that the mTOR signaling pathway is involved in the regulation of FGF23 by energy intake.

The present study confirms previous reports describing that high calorie intake results in increased FGF23 concentrations^15,16^. A problem when linking high caloric intake with FGF23 is that energy-dense diets have a high fat content and there are unavoidable collateral effects of high fat diets on the kidneys and on systemic inflammation that may result in indirect increases of FGF23. High fat diet is known to produce kidney damage^18^ and acute kidney injury is sufficient to increase FGF23 even in the absence of hyperphosphatemia^23^. High fat diets also promote inflammation^17^ which not only can contribute to kidney damage but may also have independent effects on FGF23^24^. Two recent studies have identified a link between elevations in TNFα secondary to feeding high fat and increases in FGF23^16,25^.

To further clarify the role of energy intake on FGF23 concentrations we studied energy deprived rats that were fed a diet with a very low caloric content. Energy deprived rats had reduced FGF23 concentrations when compared with energy repleted animals. To our knowledge, FGF23 has not been previously studied in animals subjected to energy deprivation. The reduction in FGF23 in calorie deprived rats reinforces the contention that energy intake directly regulates FGF23. Moreover, when data from all the animals used in the study were pooled, a highly significant correlation between energy intake and FGF23 was observed. Since elevated FGF23 concentrations are associated to cardiovascular morbidity and mortality^3–5^ reducing energy intake may have advantages on cardiovascular health that could be mediated by a decrease in FGF23.

The modulation of FGF23 concentrations by energy intake was independent of changes in intestinal P absorption and in plasma P concentrations. It is important to emphasize the quantification of absorbed P, instead of the P that was eaten because, even though efforts were made to avoid different P load, dietary modifications change feeding behaviour in rodents and thus it is difficult to adjust with precision the amount of ingested P. Moreover, it is important to evaluate absorbed P because the composition of the diet (e.g. fat content) may modify intestinal absorption of P^26^. The possibility that P intake may have influenced FGF23 by a mechanism independent of intestinal P absorption is unlikely. At any rate, if this were the case, the effect of caloric intake on FGF23 would had been underestimated since the rats that ate more P were in the LC group which also had the lower FGF23 concentrations. In conclusion, rats with equivalent P load, similar plasma P concentrations but with very different energy intake showed changes in FGF23 levels that correlated with energy intake.

In addition to P, FGF23 is hormonally regulated by calcitriol^7^. In the rats under study plasma calcitriol changed in opposite direction to plasma FGF23 and a strong inverse correlation between both parameters was identified. The regulation of calcitriol and FGF23 is bidirectional -calcitriol stimulates FGF23 production^7^ while FGF23 inhibits calcitriol synthesis^2^. Since the correlation was inverse, it seems that the changes in FGF23 were driving calcitriol production. Therefore, energy intake can modulate calcitriol production through changes in FGF23. Given the important repercussions of vitamin D on cardiovascular^27^ and bone^28^ health the indirect regulation of calcitriol, through changes in FGF23, associated to energy intake may be highly relevant.

*In vitro* studies support the effect of energy availability on FGF23 expression by bone cells. UMR 106 cells cultured in HG medium consistently expressed more mRNA FGF23 than UMR 106 cells cultured in LG medium. It is important to note that even though energy availability was limited in the LG medium, the cells cultured in LG grew almost at the same rate than the cells cultured in HG medium. In fact the time to reach confluence was not different for cells cultured in HG, 120.0 ± 0.5 hours, and in LG, 127.6 ± 4.6 hours. Thus, the cells cultured in LG were able to maintain their physiologic processes but reduced FGF23 expression. This contention was further supported by the study of osteogenic genes which were not affected by the glucose content of the culture medium.

Since HG medium has higher osmolality than LG medium, and data in the literature support the influence of osmolality on bone cell biology^29^, experiments were conducted adding mannitol to LG medium to reach the same osmolality of the HG medium. The cells cultured in LG + Man also expressed lower levels of mRNA FGF23 than the cells cultured in HG, thus ruling out any interfering effect related to changes in osmolality.

It is interesting to note that blood glucose was not altered in the *in vivo* studies, in fact plasma glucose levels were slightly lower in the rats fed HC diet. Thus, glucose, per se, is not the likely triggering factor in the response to nutrient availability. The contention that what drives the regulation of FGF23 seems to be sustained energy availability rather than glucose concentration is further supported by *in vitro* experiments in which short time changes in glucose concentration in the medium did not modify FGF23 expression.

As expected, UMR 106 cells incubated in HG increased mRNA FGF23 in response to elevated P concentrations. However, when the cells were incubated in LG they did not increase FGF23 expression even in the presence of very high P concentrations. These data are in line with previous studies that have reported that P restriction does not prevent the increase in FGF23 associated to high caloric intake^16^ and, in conjunction with the *in vivo* data reported in the present study, would argue for a preferential regulation of FGF23 by nutrient/energy availability rather than by P.

To provide a mechanistic explanation to the regulation of FGF23 by energy availability, the mTOR signaling pathway was explored, because inhibition of mTOR has been shown to mimic calorie restriction^20^. Two mTOR complexes, mTORC1 and mTORC2, are known, but only mTORC1 is rapamycin sensitive. mTORC1, which is suppressed by energy deprivation (low ATP-to-AMP ratio), is a downstream target of AMPK^30^. In an elegant study, Glosse *et al*. demonstrated that AMPK activation decreases FGF23 and that the effect of AMPK on FGF23 was mediated, at least in part, by downregulation of the calcium channel Orai1 involving store-operated calcium entry (SOCE)^14^. Both AMPK and rapamycin have an inhibitory effect on mTOR, but the fact that rapamycin by itself is able to decrease FGF23 production focus the signaling of energy-sensing that regulates FGF23 on mTOR. The involvement of the mTOR pathway was corroborated by the response to a different mTOR inhibitor, everolimus. It is interesting to note that a tendency to decreased FGF23 has also been reported in mice treated with rapamycin^31^ lending further support to the involvement of the mTOR pathway in FGF23 production.

Like AMPK, mTORC1 signaling has also been shown to enhance SOCE and mTORC1 inhibition by rapamycin has been reported to suppress STIM1, a protein necessary for SOCE activation^32^. Our data also confirm a decrease in STIM1 expression in UMR 106 cells incubated with rapamycin and everolimus. Thus, both AMPK, through orai1 inhibition, and mTORC1, through STIM1 downregulation, can lead to SOCE activation that results in a decrease in FGF23 production.

**Figure 9:**
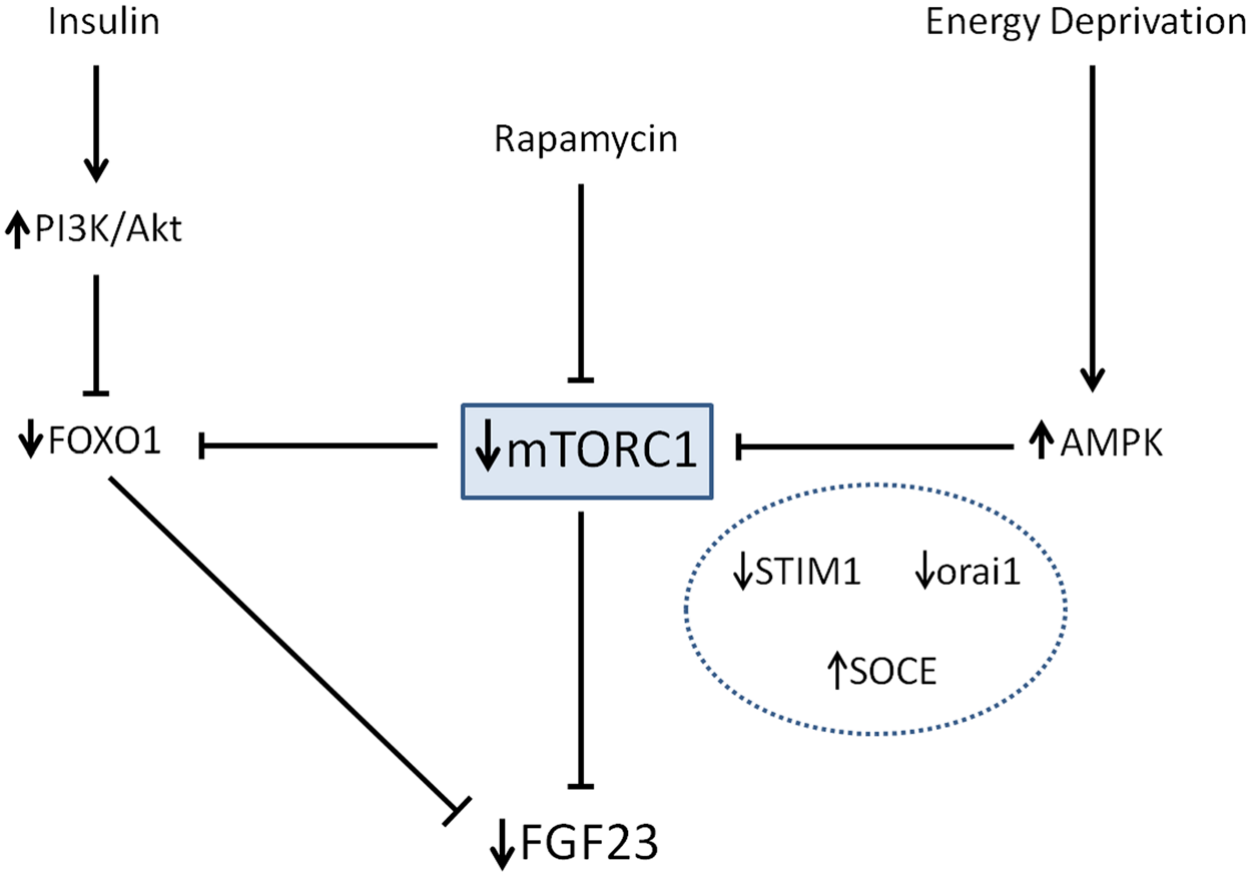
Proposed model integrating the actions of energy deprivation, rapamycin and insulin on FGF23 production. Energy deprivation stimulates AMPK and, like rapamycin, inhibits mTORC1. The increase in AMPK and the decrease in mTORC1 reduce FGF23 through an increase in SOCE mediated by down regulation of STIM1 and orai1. Both inhibition of mTORC1 and insulin signaling through PI3K/Akt inhibit FOXO1 and this also decreases FGF23 production.

Bar *et al*. have reported that insulin suppresses the production of FGF23 by inhibition of FOXO1 transcription factor^13^, and these results have been reproduced in our laboratory (data not shown). Since insulin is known to inhibit AMPK activity^33^ the data from Bar *et al*. and Glosse *et al*. could be considered contradictory. However, our results showing involvement of the mTOR pathway may help to harmonize the information provided by these two studies. Both insulin signaling, through the phosphatidylinositol 3-kinase (PI3K/Akt) pathway, and mTOR inhibition by rapamycin (or by energy restriction) are able to inactivate FOXO1^13,34^. Thus, as shown in Fig. 9, we propose that mTOR is a central molecule in the regulation of FGF23 by energy availability since it integrates two signaling pathways which are dependent on insulin and energy availability, respectively.

In conclusion, this study shows a direct regulation of FGF23 production, both *in vivo* and *in vitro*, by energy availability and demonstrates that the mTOR signaling pathway plays a central role in this regulatory system.

## Material and Methods

### Ethics

All experimental protocols were reviewed and approved by the Ethics Committee for Animal Research of the University of Cordoba and by Junta de Andalucia (Spain) (Ethical Code Number 30/10/2017/148, date 08/11/2017). All protocols were carried out in accordance with the approved guidelines. They followed the guiding principle laid down by the Higher Council of Scientific Research of Spain following the normal procedures directing animal welfare and adhered to the recommendations included in the Guide for Care and Use of Laboratory Animals (US Department of Health and Human Services, NIH) and European laws (Art. 41.1, Real Decreto 53/2013, 01/02 Dec 2012/707/UE) and regulations on protection of animals, under the advice of specialized personnel.

### *In vivo* experiments

#### Animals and diets

Female Wistar rats, aged 2 months at the beginning of the studies, were used in the experiments. Rats were provided by the Animal Housing Facilities of the University of Cordoba (Cordoba, Spain) and were housed with a 12 h/12 h light/dark cycle. Appropriate measures were taken to ensure animal welfare and to address the basic behavioral and physiological needs of rats.

Three diets were used in the experiments: a) diet with normal caloric (NC) content, Metabolizable Energy = 3518 kcal/kg, (Altromin C 1090-10, AltrominSpezialfutter GmbH, Germany); b) diet with high caloric (HC) content, Metabolizable Energy = 5241 kcal/kg (Altromin C 1090-60, AltrominSpezialfutter GmbH, Germany); and c) diet with low caloric (LC) content, Metabolizable Energy = 1314 kcal/kg (Altromin C 1012, AltrominSpezialfutter GmbH, Germany). All diets contained normal amounts of calcium (Ca), P and vitamin D: 0.6% of Ca, 0.6% of P and 500 IU/g of vitamin D.

#### Experimental design

Rats were allotted to 3 groups (n = 9 per group) that received the study diets for 30 days. Rats in Group 1 (Control) were fed NC diet ad libitum. Rats in Group 2 received HC diet ad libitum. Rats in Group 3 were fed LC diet. To prevent overeating, the amount of food made available to the rats on the LC diet was adjusted to achieve a caloric intake that was reduced by 35% when compared with the NC group.

During the last week of the study the rats were housed in metabolic cages, allowing daily control of food and water intake and collection of feces. At the end of the experiments, rats were sacrificed by exsanguination under general anesthesia (inhaled isoflurane).

#### Blood chemistries

Blood was collected from the abdominal aorta at the time of sacrifice. Blood glucose was measured immediately after collection with a blood glucose meter (Bayer Consumer Care AG, Basel, Switzerland). Afterwards, plasma was separated by centrifugation and stored at −20 °C until assayed. Plasma concentrations of P were measured by spectrophotometry (BioSystems SA, Barcelona, Spain). ELISA tests were used to quantify intact plasma FGF23 (Kainos Laboratories, Tokyo, Japan). Radioimmunoassay (Immunodiagnostic Systems Ltd, Boldon, UK) was used in plasma samples to determine 1,25-dihydroxyvitamin D (calcitriol).

#### Fecal chemistries

Fecal samples were dried, ashed and demineralized with 0.6 mmol/l HNO_3_ solution. Fecal P was measured by inductively coupled plasma mass spectrophotometry (ICP-MS, Perkin Elmer Elan DRC-e, Walthan, Massachusetts, USA). Net intestinal absorption of P was calculated as follows:

$${\rm{P}}\,{\rm{net}}\,{\rm{absorption}}\,({\rm{mg}}/{\rm{day}})={\rm{P}}\,{\rm{intake}}-{\rm{P}}\,{\rm{fecal}}\,{\rm{excretion}}$$

### *In vitro* experiments

#### Cell culture

Rat osteosarcoma cell line UMR 106 (ATCC, Manassas, VA, USA) was used for all *in vitro* experiments. UMR 106 cells were cultured in Dulbecco’s modified Eagle’s medium (DMEM) (Sigma-Aldrich, St. Louis, MO, USA) supplemented with 10% fetal bovine serum (FBS) (Biowest, Riverside, MO, USA), 100 mg/l streptomycin (Normon, Madrid, Spain), 100 IU/ml penicillin (ERN, Barcelona, Spain), 0.29 mg/l fungizone (Teva, Madrid, Spain), 2 mM ultra-glutamine (Lonza, Walkersville, MD, USA) and 1 mM sodium pyruvate (Lonza, Walkersville, MD, USA) at 5% CO_2_, 37 °C temperature and water saturated atmosphere. Cells were seeded in 6 wells plates with 10,000 cells/cm^2^ and maintained in DMEM up to 90% confluence. Once confluence was reached, as UMR 106 cells do not easily express FGF23, FGF23 production was stimulated by adding calcitriol (10^−8^ M) (Kern pharma, Barcelona, Spain) for twenty-four hours as previously reported^13^. Culture medium was changed every other day. Cells in passage six were used for all experiments. To study the effect of energy availability, cells were incubated until they reached confluence in either DMEM with high glucose (HG, 4.5 g/l), DMEM with intermediate glucose (2 g/l) or DMEM with low glucose (LG, 1 g/l). Further studies were carried out in which cells that had grown in HG medium were exposed to LG medium for shorter periods of time. Additional experiments were performed to assess the influence of osmolality. In these studies, mannitol (Man, 19.42 mOsmol/l) (Fresenius Kabi España, Barcelona, Spain) was added to the LG medium to achieve the same osmolality the HG medium. To evaluate the effect of P, both HG and LG groups were incubated for 6 days with standard DMEM (NP, 1 mM) or with DMEM with high P concentration (HP, 4 mM) in which PO_4_HNa_2_/PO_4_H_2_Na (Sigma-Aldrich, St. Louis, MO, USA) was added in a ratio 1/2. To explore the involvement of the mTOR signaling pathway 5 nM and 10 nM rapamycin (Rap) (Calbiochem, San Diego, CA, USA) was added to the HG medium and Rap was maintained in the medium during the 6 days that lasted the experiment. Since Rap needs to be dissolved in dimethyl sulfoxide (DMSO), an additional control group was included. In this group (HG + DMSO) the HG culture medium contained the same amount of DMSO, 0.009%, (Sigma-Aldrich, St. Louis, MO, USA) used to dissolve Rap. Further studies were carried out with cells grown in HG medium that were exposed to rapamycin for shorter periods of time. Additional experiments were performed adding 10 nM everolimus (Sigma-Aldrich, St. Louis, MO, USA) to cells cultured in HG medium.

#### RNA extraction and real time reverse transcription-polymerase chain reaction (RT-PCR)

Total RNA was isolated using TRIzol reagent protocol (Invitrogen) and a treatment with DNase I amplification Grade (Sigma-Aldrich, St. Louis, MO, USA) was done according to the manufacturer’s instruction. Quantification was performed by spectrophotometry (ND-1000, Nanodrop Technologies, Wilmington, DE, USA). The sequence of primers used for RT-PCR is shown in Table 1. Quantification was done using the QuantiTect SYBR Green RT-PCR kit (Qiagen GmbH, Hilden, Germany) for 50 µg of RNA and 1 µl of primer. The mRNA expression was analyzed in the Light Cycler thermal cycler system (Roche Diagnostics, Indianapolis, IN, USA) and the relative expression of the target genes was determined using the 2^−ΔΔCt^ method.

**Table 1:** Sequences of the primers used for real-time RT-PCR.

Gene	Forward primer (5′-3′)	Reverse primer (5′-3′)
FGF23	TGGCCATGTAGACGGAACAC	GGCCCCTATTATCACTACGGAG
RunX2	CGGGAATGATGAGAACTACTC	GCGGTCAGAGAACAAACTAGGT
Osterix	GTACGGCAAGGCTTCGCATCTGA	TCAAGTGGTCGCTTCGGGTAAAG
STIM1	CAGTACTACAACATCAAGAAGC	TTTTTATTTTCTCAGCCCCC
Tbp	ACTCCTGCCACACCAGCC	GGTCAAGTTTACAGCCAAGATTCA

All primers were purchased from Eurofins Genomics Germany GmbH, Ebersberg, Germany except STIM 1 which was purchased from Sigma-Aldrich, St. Louis, MO, USA.

FGF23, fibroblast growth factor 23; RunX2, Runt-related transcription factor 2; STIM1, Stromal interaction molecule 1; Tbp, TATA sequence binding protein.

### Statistics

Values are expressed as the mean ± standard error (SE). The difference between means for two different groups was determined by t-tests; the difference between means for three or more groups was assessed by ANOVA. Fisher LSD test was used as a post-hoc procedure. A correlation study was carried out using the Pearson test. A p < 0.05 was considered significant. Statistical analysis was performed using GraphPad Prism version 6.01 software.

## Supplementary information

Supplementary Figure 1.

## Data Availability

The datasets generated during and/or analysed during the current study are available from the corresponding author on reasonable request.
