# Supplementary material for: Direct regulation of fibroblast growth factor 23 by energy intake through mTOR
Source: Sci Rep. 2020 Feb 4;10:1795. doi: 10.1038/s41598-020-58663-7 (PMC7000745; doi:10.1038/s41598-020-58663-7)
Supplement: Supplementary file 1 — Supplementary Figure 1. [file 41598_2020_58663_MOESM1_ESM.pdf]

Direct regulation of fibroblast growth factor 23 by energy intake through mTOR

Angela Vidal[1,2], Rafael Rios[1,2], Carmen Pineda[1,2], Ignacio Lopez[1,2], Juan R. Muñoz-Castañeda[2], Mariano Rodriguez[2], Escolastico Aguilera-Tejero[1,2,*,+], Ana I. Raya[1,2,+]

[1]*Department of Animal Medicine and Surgery, University of Cordoba, Campus Universitario Rabanales, Cordoba, Spain*
[2]*Maimonides Biomedical Research Institute of Cordoba (IMIBIC), Reina Sofia University Hospital, University of Cordoba, Cordoba, Spain*

*Corresponding author. Correspondence and requests for materials should be addressed to Escolastico Aguilera-Tejero (E A-T). Dept. Medicina y Cirugia Animal, University of Cordoba. Ctra. Madrid-Cadiz, Km 396, 14014, Cordoba, Spain
E-mail: eaguilera@uco.es

[+]These authors contributed equally to this work.

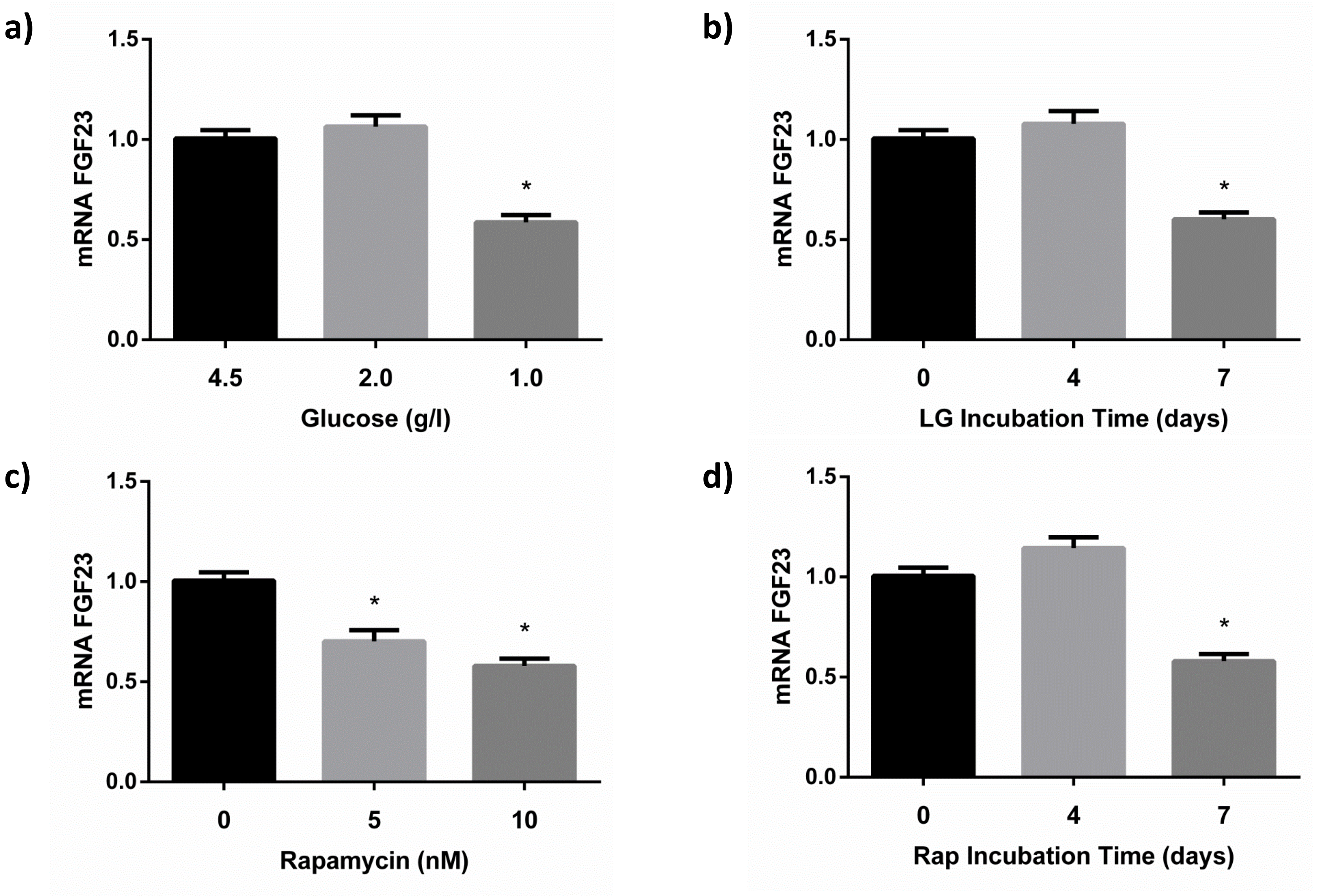

Supplementary Figure 1. a) Dose response of mRNA FGF23 production by UMR 106 cells to incubation for 6 days with different concentrations of glucose. b) Time dependence of mRNA FGF23 production by UMR 106 cells incubated with low glucose (LG, 1 g/l). c) Dose response of mRNA FGF23 production by UMR 106 cells to incubation for 6 days with different concentrations of rapamycin. d) Time dependence of mRNA FGF23 production by UMR 106 cells incubated with rapamycin (Rap, 10 nM).
